# Frequent origins of traumatic insemination involve convergent shifts in sperm and genital morphology

**DOI:** 10.1002/evl3.268

**Published:** 2021-12-30

**Authors:** Jeremias N. Brand, Luke J. Harmon, Lukas Schärer

**Affiliations:** ^1^ Department of Environmental Sciences, Zoological Institute University of Basel Basel CH‐4051 Switzerland; ^2^ Department of Tissue Dynamics and Regeneration Max Planck Institute for Biophysical Chemistry Göttingen DE‐37077 Germany; ^3^ Department of Biological Sciences University of Idaho Moscow Idaho 83843

**Keywords:** Copulatory wounding, correlated evolution, evolution, female genitalia, hypodermic insemination, parallel evolution, phylogenetics, sexually antagonistic coevolution, traumatic mating

## Abstract

Traumatic insemination is a mating behavior during which the (sperm) donor uses a traumatic intromittent organ to inject an ejaculate through the epidermis of the (sperm) recipient, thereby frequently circumventing the female genitalia. Traumatic insemination occurs widely across animals, but the frequency of its evolution, the intermediate stages via which it originates, and the morphological changes that such shifts involve remain poorly understood. Based on observations in 145 species of the free‐living flatworm genus *Macrostomum*, we identify at least nine independent evolutionary origins of traumatic insemination from reciprocal copulation, but no clear indication of reversals. These origins involve convergent shifts in multivariate morphospace of male and female reproductive traits, suggesting that traumatic insemination has a canalizing effect on morphology. We also observed sperm in both the sperm receiving organ and within the body tissue of two species. These species had intermediate trait values indicating that traumatic insemination evolves through initial internal wounding during copulation. Finally, signatures of male‐female coevolution of genitalia across the genus indicate that sexual selection and sexual conflict drive the evolution of traumatic insemination, because it allows donors to bypass postcopulatory control mechanisms of recipients.

**impact summary**

Traumatic insemination is an extreme type of mating behavior. Instead of copulation, traumatically mating species transfer sperm, and potentially other substances, directly through the partner's skin. Sometimes, they inject sperm into the female genitals, but in other cases simply into body tissue. Injected sperm then actively move through the tissue, eventually fertilizing the partner's eggs. Traumatic insemination occurs in many invertebrate animals such as insects, snails, and flatworms. However, in most studied systems, it has evolved only a few times, making it difficult to determine the general adaptations required for the behavior. Here, we study traumatic insemination in *Macrostomum* flatworms. Based on observations of received sperm and reproductive morphology, we show that traumatic insemination has evolved many times. Intriguingly, we do not find any evidence for a reversal back to copulation once traumatic insemination arises. And we see that the evolution of traumatic insemination coincides with striking changes across all our measured traits. Therefore, traumatic insemination could be an evolutionary one‐way street leading to irreversible morphological changes. We also observed coevolution between male and female genitals in copulating species, suggesting that traumatic insemination could be an alternative strategy in a coevolutionary chase between mating partners. In particular, it could allow the sperm donor to force mating or bias sperm competition and cryptic female choice in their favor.

The sexes frequently show differences in mating propensity because male fertility (i.e., fertilized egg production) is often limited by the number of matings a male achieves, whereas female fertility is often limited by the amount of resources a female invests into eggs and offspring (Bateman 1948; Arnold 1994; Janicke et al. 2016). The resulting conflict over mating rate has far‐reaching consequences, often resulting in “Darwinian sex roles” with choosy females and eager males (Parker 2014). Females may benefit from choice by selecting males based on genetic compatibility, genetic quality (Puurtinen et al. 2009), and/or direct benefits (e.g., nuptial gifts; Arnqvist and Nilsson 2000). Evidence for female choice is widespread and there are many species where females mate multiply, suggesting polyandry may indeed result in such benefits (Hosken et al. 2003). However, females may also mate multiply as a result of male harassment, and although that could be costly to females, resisting male harassment might be even costlier (Hosken et al. 2003; Arnqvist and Rowe 2005). Costly harassment is expected to arise frequently, because female choice necessarily goes against the rejected males’ interests (Parker 2006), potentially leading to sexually antagonistic coevolution between male persistence and female resistance traits (Rice 1996; Arnqvist and Rowe 2002a, 2005).

In polyandrous species, sexual selection and sexual conflict continue after copulation through intricate interactions of the female genital tract with the male intromittent organs and the received ejaculate (Charnov 1979; Birkhead and Pizzari 2002; Wedell et al. 2010). Female genitalia might exert postcopulatory control through differential sperm storage, sperm ejection, or sperm digestion, thus applying selective filters on male genital and ejaculate traits. In analogy to the precopulatory conflict, it is then possible for traits in males to arise that attempt to bypass or influence the female‐choice and resistance mechanisms, again resulting in sexually antagonistic coevolution (Charnov 1979; Birkhead and Pizzari 2002; Wedell et al. 2010).

Such coevolution can drive the emergence of male traits that inflict considerable harm on females (Arnqvist and Rowe 2002a; Morrow and Arnqvist 2003; Morrow et al. 2003). A striking example that implicates such harm is traumatic insemination, which occurs in some internally fertilizing species and involves the infliction of a wound to the female's integument through which the male then transfers its ejaculate (Lange et al. 2013). Because traumatic insemination occurs in both gonochoristic (separate‐sexed) and hermaphroditic species (Lange et al. 2013), we in the following use the more general terms (sperm) donor and (sperm) recipient to refer to the two sexual roles, with no loss of generality (Schärer et al. 2015).

Although traumatic insemination often results in costs to recipients (Morrow and Arnqvist 2003; Reinhardt et al. 2003; Benoit et al. 2012; Lange et al. 2013; Reinhardt et al. 2015a; Tatarnic 2018), it has evolved independently in many invertebrate phyla, including Callimorpha, Arthropoda, Annelida, Gastrotricha, Gnathostomulida, Mollusca, Nematoda, Platyhelminthes, and Rotifera (see Lange et al. 2013). And although natural selection might play a role in some taxa—especially the endoparasitic Strepsiptera (Tatarnic et al. 2014; Kathirithamby et al. 2015)—it likely often evolves due to sexual selection and sexual conflict. Specifically, traumatic insemination can enable donors to force copulation and thus minimize the control that the recipient could otherwise exert over mating (Morrow and Arnqvist 2003). It may also allow the donor to bypass the recipient's genitalia, by depositing sperm either closer to the site of fertilization (Kathirithamby et al. 2015; Peinert et al. 2016) or even directly within the relevant tissue (Stutt and Siva‐Jothy 2001; Morrow and Arnqvist 2003), thus reducing the recipient's ability to control the fate of the received ejaculate (Charnov 1979; Lange et al. 2013). In this view, traumatic insemination allows the donor to bypass the influence of the recipient's sexually antagonistic choice and resistance mechanisms, temporarily gaining an advantage in the coevolutionary chase.

However, because conflicts persist under traumatic insemination, we expect selection to then act on traits that allow the recipient to regain control over mating and/or the fate of the received ejaculate. For example, some species of bed bugs have evolved what is considered a secondary vagina, a structure shown to reduce the costs incurred due to traumatic insemination (Reinhardt et al. 2003; Siva‐Jothy 2006). But even without the emergence of new organs, recipients could evolve behavioral or physiological responses to avoid traumatic insemination (such as parrying strikes during penis fencing in polyclad flatworms; Michiels and Newman 1998) or to manipulate and control the hypodermically received ejaculate (e.g., similar to sperm digestion in copulating species; Sluys 1989; Koene 2006; Koene et al. 2009).

Besides bypassing recipient choice and resistance mechanisms, traumatic insemination could also evolve due to sperm competition. In many internally fertilizing species, sperm of unrelated donors compete within the female genital tract for fertilization of the recipient's eggs (Parker 1998). In this context, traumatic insemination might allow donors to avoid sperm competition and prevent competing donors from removing their previously donated sperm, resulting in paternity benefits (Lange et al. 2013). Indeed, traumatic insemination seems to affect sperm competition in a family of spiders, where sperm precedence is biased toward the first male in a species with traumatic insemination, whereas it is biased toward the second male in its nontraumatically mating relatives (Milan 2009). In contrast, traumatic insemination is associated with last male precedence in one species of bed bug (Stutt and Siva‐Jothy 2001), so its effects on sperm competition might depend on a species’ morphology and ecology.

Traumatic insemination might evolve more frequently in hermaphrodites due to sexual conflict over the mating roles (Charnov 1979; Michiels 1998; Anthes et al. 2006; Anthes 2010; Schärer et al. 2015). In general, and analogous to the situation outlined for gonochorists (Bateman 1948), a hermaphrodite already carrying enough received sperm to fertilize its own eggs might gain little from additional matings as a recipient, whereas it could still gain additional fertilizations by acting as a donor (Charnov 1979). It is thus likely that, on average, individual hermaphrodites show a preference for sperm donation (Charnov 1979; Michiels 1998; Anthes et al. 2006; Anthes 2010; Schärer et al. 2015) and this rationale is supported by several laboratory studies (Anthes et al. 2006, 2010; Pélissié et al. 2012). Traumatic insemination then potentially allows individuals to attempt unilateral enforcement of donation while avoiding receipt. Additionally, hermaphrodites may engage in harmful matings more readily, because any fitness costs an individual incurs as a recipient may be partially compensated by fitness benefits it incurs as a donor (Michiels 1998; Michiels and Koene 2006). Indeed, 11 out of 23 well‐supported independent origins of traumatic insemination occurred in hermaphrodites (Lange et al. 2013), even though hermaphrodites amount to only ∼6% of animals (Jarne and Auld 2006). Hermaphrodites are thus ideal study organisms for investigations of traumatic insemination, because—although it has been studied in some charismatic systems (Michiels and Newman 1998; Morrow and Arnqvist 2003; Kamimura 2007; Tatarnic and Cassis 2013; Peinert et al. 2016)—we currently still know little about the frequency and consequences of its evolution (Lange et al. 2013; Reinhardt et al. 2015a; Tatarnic 2018).

Here, we present comparative work on the evolution of traumatic insemination across the genus *Macrostomum*, a species‐rich taxon of hermaphroditic free‐living flatworms. In *Macrostomum*, traumatic insemination is called hypodermic insemination (HI), because in several species the donor uses a needle‐like stylet (Fig. 1A) to inject sperm through the mating partner's epidermis. Sperm then move through the recipient's body to the site of fertilization, but the precise location and mechanism of fertilization is currently unknown (Schärer et al. 2011; Ramm et al. 2012, 2015). Injected sperm can often be observed inside the parenchymal tissues of these highly transparent animals (Schärer et al. 2011; Ramm et al. 2012, 2015; Winkler and Ramm 2018), making it feasible to screen a large number of species for convergent evolution of HI. And while we here present evidence that not all traumatically mating *Macrostomum* species may inject sperm through the external epidermis, we nevertheless use the term HI for consistency with previous literature.

**Figure 1:**
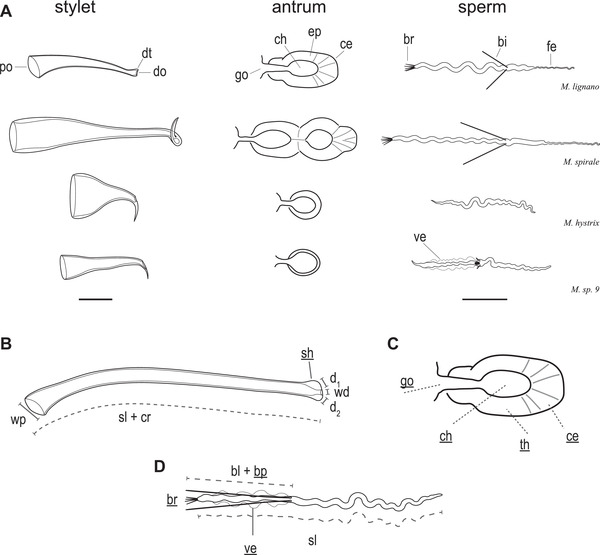
(A) Representative drawings of the morphology of the stylet (male intromittent organ), the antrum (female reproductive organ), and the sperm of four *Macrostomum* species. The well‐studied model *M. lignano* represents the typical morphology for reciprocally mating species, showing a stylet with wide proximal opening (po), blunt distal thickenings (dt) on the distal opening (do) and a complex sperm with an anterior feeler (fe), two stiff lateral bristles (bi), and a terminal brush (br). Moreover, the antrum has a single genital opening (go), and a single chamber (ch) with a thickened antrum epithelium (ep) and a cellular valve (ce). *Macrostomum spirale* also mates reciprocally, but compared to *M. lignano* the stylet has a sharp distal thickening; however, the antrum is more elaborate with multiple thickened antrum chambers. *Macrostomum hystrix* and *Macrostomum* sp. 9 represent two convergent origins of hypodermic insemination in the reciprocal clade. Their stylets show a characteristic highly asymmetric and sharp distal thickening, and they have a simple antrum with a thin antrum epithelium and simple sperm without a brush. However, *Macrostomum* sp. 9 additionally has reduced sperm bristles and a thin sperm velum (ve). Note that, given the striking diversity across the *Macrostomum* genus, it is not possible to clearly delimit all the sperm traits originally defined in *M. lignano* in some of the species. (B–D) Schematic representations of the stylet, antrum, and sperm traits we measured here (for details see SI Morphology). Traits scored categorically on a per species basis are underlined. (B) Stylet measures include stylet length (sl), stylet curviness (cr), width of the proximal opening (wp), width of the distal opening (wd), stylet sharpness (sh), and distal asymmetry calculated as the difference between length d_1_ and d_2_. (C) Antrum measures include number of genital openings (go), antrum thickness (th), presence and thickness of an anterior cellular valve (ce), antrum chamber complexity (ch), and a compound measure of antrum complexity (combining scores for ch, th, and ce). (D) Sperm measures include sperm length (sl), sperm bristle length (bl), presence of a sperm brush (br), presence of a sperm velum (ve), and presence of the sperm bristle (bp). Antrum drawings are adapted from Janssen et al. (2015) and used with permission.

The genus comprises two phylogenetically well‐separated clades (Brand et al. 2022), a “hypodermic clade” members of which are thought to exclusively mate through HI and a “reciprocal clade” in which most species mate reciprocally (called Clade 1 and 2, respectively, in Schärer et al. 2011), with the latter containing a convergent origin of HI in *M. hystrix* (Schärer et al. 2011). During reciprocal copulation two worms insert their—often relatively blunt—stylet (Fig. 1A) via their partner's female genital opening into the female sperm storage organ, the female antrum (further called antrum), so that both can donate and receive sperm in the same mating (Schärer et al. 2004). Many reciprocally copulating species perform a postcopulatory suck behavior, where worms place their mouth over their own female genital opening and suck, presumably in an attempt to remove components of the received ejaculate from their antrum (Schärer et al. 2004, 2011, 2020; Vizoso et al. 2010; Singh et al. 2020). This ejaculate removal could target manipulative seminal fluids, since the ejaculate of the model species *M. lignano*, contains substances affecting the mating partner's propensity to perform the suck behavior (Patlar et al. 2020; Weber et al. 2020). Alternatively, the suck behavior could also reduce the number of stored sperm (e.g. to lower the risk of polyspermy), constitute a form of cryptic female choice (e.g. to favor donors of higher quality), and/or represent a resistance trait in sexual conflict over mating roles (i.e. to undo unwanted sperm receipt, Vizoso et al. 2010; Schärer et al. 2011).

If the suck behavior is a recipient resistance trait, we might expect the evolution of donor persistence traits, potentially leading to antagonistic coevolution (Arnqvist and Rowe 2005). Indeed, the sperm of reciprocally copulating species generally have a thin anterior feeler and two stiff lateral bristles that could represent such persistence traits (Fig. 1A), serving to anchor the sperm in the antrum to prevent removal during the suck behavior (Vizoso et al. 2010; Schärer et al. 2011). In contrast, sperm of species with HI (i.e. the hypodermic clade and *M. hystrix*) lack these bristles and have a simplified morphology, presumably because they no longer need to resist the suck behavior (Vizoso et al. 2010; Schärer et al. 2011), which has so far never been observed in species with HI. These sperm may instead be adapted to efficiently move through the partner's tissues (Fig. 1A), and one such adaptation could hypothetically also include a reduced sperm size (Schärer et al. 2011). Moreover, species with reciprocal copulation have an antrum with a thickened epithelium and an anterior cellular valve (a specialized epithelium through which eggs enter the antrum, Vizoso et al. 2010; Fig. 1A) that interacts with the sperm feelers. But species with HI have a simple antrum lacking an evident cellular valve, presumably because it no longer interacts with the donor's stylet and sperm, and instead is used for egg‐laying only (Schärer et al. 2011). Based on these findings, the observed adaptations to reciprocal copulation and HI have been described as the reciprocal and hypodermic mating syndrome, respectively, since they each constitute specific combinations of morphological (sperm, stylet and antrum) and behavioral traits (Schärer et al. 2011).

If HI allows donors to bias the sexual conflicts over mating roles in their favor, e.g. by offering a more effective route to obtain male fitness, then we would expect it to evolve frequently. But it is currently unclear whether HI has convergently arisen more than once within the reciprocal clade. It is also unclear if such transitions are reversible or if the emergence of HI alters the coevolutionary dynamics between donor and recipient, so that species cannot readily revert to reciprocal copulation. Here we combine morphological information on 145 *Macrostomum* species with a recent phylogenomic analysis of the genus (Brand et al. 2022), to identify additional independent origins of HI and to describe convergent changes in sperm design and genital morphology that accompany its evolution. Using ancestral state reconstruction (ASR), we further ask whether species can revert to reciprocal copulation once HI has arisen. Finally, we test for the covariation between male and female genital traits that is expected if sexually antagonistic coevolution drives the emergence of HI.

## Materials and Methods

### SPECIES COLLECTED AND PHYLOGENETICS

**Figure 2:**
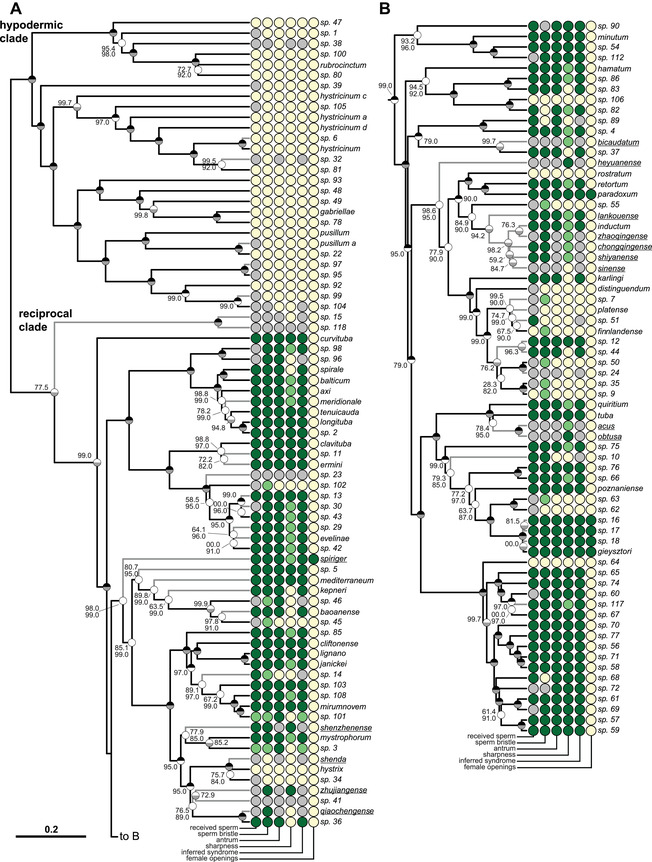
Phylogeny of the genus *Macrostomum*, showing the states of six reproductive traits. The ultrametric phylogeny (C‐IQ‐TREE) includes all 145 species from (Brand et al. 2022). Branch supports are ultrafast bootstraps (top, black if 100) and approximate likelihood ratio tests (bottom, gray if 100). Species without available transcriptomes that were added based on a *28S rRNA* fragment are indicated with gray branches. Underlined species names indicate that the trait scoring is based on information from the literature. Two phylogenetically well‐separated clades are labeled in panel A. Members of the “hypodermic clade” are thought to exclusively mate through hypodermic insemination (HI), whereas those of the “reciprocal clade” primarily mate reciprocally. Columns indicate the states of six reproductive traits from light to dark (i.e., yellow, light green, and dark green for trinary states; or yellow and dark green for binary states; gray indicates missing data): received sperm location (hypodermic, both, in antrum), sperm bristle state (absent, reduced, present), antrum state (simple, thickened), sharpness of stylet (sharp, neutral, blunt), inferred mating syndrome (hypodermic, intermediate, reciprocal), and the number of female openings (one, two). The phylogeny is split into two parts (panels A and B) for visualization. See also Figure S2 combining this figure with drawings of the stylet and sperm morphology available from Brand et al. (2022).

We used phylogenetic information and operational species assignments that we recently generated by integrating morphological and transcriptome data, supplemented with partial *28S rRNA* sequences and information from the literature (Brand et al. 2022). We used a maximum‐likelihood based phylogeny including 145 species (C‐IQ‐TREE, shown in Fig. 2), based on a concatenated multiple sequence alignment of 385 orthologous proteins (94,625 amino acid positions with 22.9% missing data) from 98 species, and supplemented with partial *28S rRNA* sequences, allowing the addition of 47 species (Brand et al. 2022). To assess how sensitive our analyses are to phylogenetic uncertainty, we repeated all analyses with two alternative phylogenies, including only the 98 species with full transcriptome information and inferred using maximum‐likelihood (H‐IQ‐TREE) or Bayesian methods (H‐ExaBayes). Since all results were quantitatively similar and qualitatively identical, we focus on the C‐IQ‐TREE results, but report the additional analyses in the supplementary files.

### MORPHOLOGICAL DATA

We previously sampled *Macrostomum* species across the globe and deposited extensive image and video documentation of these primarily field‐collected specimens (Schärer et al. 2020; Brand and Schärer 2021; Brand et al. 2022). Here we used this raw material to extract both quantitative (Q) and categorical (C) data from 1442 of these deposited specimens. We then supplemented the data based on taxonomical descriptions of a few species we did not collect ourselves. Categorical data were determined on a per species basis, while quantitative data were taken per individual. We measured body size (Q, 1380 specimens, mean per species: 11, range per species: 1–47) as the total body area and either measured or scored various aspects of the stylet, of the sperm, and of the antrum (see Fig. 1B–D for a schematic representation of the measured traits and see SI Morphology and Tables A1 and A2 and Figs. A1–A3 therein for more details). Specifically, for the stylet we measured the stylet length (Q, 949 specimens, mean: 7.8, range: 1–39), the stylet curviness (Q, 956 specimens, mean: 7.8, range: 1–39), the width of the proximal stylet opening (Q, 1053 specimens, mean: 8.6, range: 1–39), the width of the distal stylet opening (Q, 1043 specimens, mean: 8.5, range: 1–39), the degree of asymmetry of the distal thickening (Q, 936 specimens, mean: 7.7, range: 1–39), and scored the sharpness of the distal thickening (C). For the sperm, we measured the sperm length (Q, 1765 sperm of 504 specimens, mean: 15.2 sperm of 4.3 specimens, range: 1–108 sperm of 1–26 specimens), the bristle length (Q, 1021 sperm of 294 specimens, mean: 12.6 sperm of 3.6 specimens, range: 1–55 sperm of 1–12 specimens), the sperm bristle state (C), the presence of a brush (C), and the presence of a velum (C). Finally, for the antrum we scored the number of genital openings (Q), the antrum thickness (C), the presence and thickness of the anterior cellular valve (C), the antrum chamber complexity (C), and an overall compound measure of antrum complexity (C). Morphometric analyses were performed using ImageJ (Rueden et al. 2017, version 1.51w) and the plugin ObjectJ (version 1.04r, available at https://sils.fnwi.uva.nl/bcb/objectj/). The pixel length of structures was converted into micrometer using a stage micrometer. For comparative analysis, we transformed body area (log_10_ of the square‐root) and all linear measures (log_10_ of stylet length, width of the proximal opening, width of the distal opening, degree of asymmetry of the distal thickening, sperm length, and bristle length). See Table S1 for sample sizes, Table S2 for all measurements, and Table S3 for species mean values.

### INFERRED MATING SYNDROME

The original definition of the mating syndromes integrated morphological and behavioral traits (Schärer et al. 2011), but because we lacked behavioral data for most species, we adapted these definitions, relying instead on several morphological traits and the observed received sperm location to derive the inferred mating syndrome (Table 1; see also SI Morphology). We assigned species to the hypodermic mating syndrome if we exclusively found hypodermic received sperm, because this represents strong evidence for HI, as opposed to species where we observed both hypodermic sperm and received sperm in the female antrum, which we classified as intermediate (Table 1). Moreover, because hypodermic sperm can be difficult to observe, especially in species with low investment into sperm production, we also assigned species that lacked received sperm observations to the hypodermic mating syndrome based on their morphology alone, namely, when they had a simple antrum, a sharp stylet, and absent or reduced sperm bristles (Table 1). And although observing received sperm in the female antrum may not exclude occasional HI, it is a strong indication of the reciprocal mating syndrome, especially when it occurs in a species with a blunt stylet. We, therefore, assigned all species with received sperm in the antrum and a blunt stylet to the reciprocal mating syndrome (Table 1). Because some reciprocally mating species also have a sharp stylet (e.g., *M. spirale*), which could possibly wound the partner internally during mating (pers. obs.), we also assigned these species to the reciprocal mating syndrome, provided that we observed received sperm in the antrum, and that they had sperm with bristles (Table 1). These assignments based on morphology alone are supported by our analysis of correlated evolution, showing a strong association between the received sperm location and both sperm bristle state and antrum type, respectively (see *Results*). The inferred mating syndrome is therefore a more inclusive classification of HI compared to an assignment based on received sperm location alone.

**Table 1:** Assignment of the inferred mating syndrome based on different reproductive traits. Species were assigned to an inferred mating syndrome based on the location of received sperm in the body (antrum, in the antrum only; hypodermic, hypodermic only; both, in the antrum and hypodermic; NA, no observation), the sperm bristle state (absent, reduced or present), the antrum state (simple or thickened), and the shape of the distal thickening of the stylet (sharp or blunt). Twenty‐six species with either not enough (22 species) or contradictory (four species) information were not assigned to a syndrome. Note, that all 24 species with only hypodermic sperm had the same morphological states, but this was not a condition for their assignment (hence the brackets). Similarly, all 69 species assigned to the reciprocal mating syndrome had a thickened antrum, but this was also not a condition for their assignment. See also *Materials and Methods*

	Received Sperm Location				Morphology			
Syndrome	Antrum	Hypodermic	Both	NA	Sperm bristle	Antrum	Stylet	*N*
Hypodermic		24			(Reduced/absent)	(Simple)	(Sharp)	24
Hypodermic				18	Reduced/absent	Simple	Sharp	18
Intermediate			2		Reduced	Thickened	Sharp	2
Reciprocal	61			6	Any state	(Thickened)	Blunt	67
Reciprocal	8				Present	(Thickened)	Sharp	8
Unclear	7			19	Other combinations			26

### CORRELATED EVOLUTION

Because we do not have direct observations of received sperm in all species, we first conducted a correlation test between sperm bristle state and received sperm location, and then tested for correlated evolution between both of these variables and the antrum type. We scored all traits as binary and applied Pagel's correlation test (Pagel 1994) as implemented in BayesTraits3 (available at http://www.evolution.rdg.ac.uk/BayesTraitsV3.0.2/BayesTraitsV3.0.2.html). For each pair of traits, we only included species with complete information on both traits. We ran four independent MCMC chains for 510 million iterations with a burn‐in of 10 million iterations and retaining every 1000th iteration. Marginal likelihood was calculated using stepping‐stones with 1000 power posteriors estimated with 10,000 iterations each. We assessed convergence using Gelman's *R* implemented in the coda R package (Plummer et al. 2006) and upon confirming convergence merged the chains for further analysis. Models were compared with Bayes factors using the marginal likelihoods (i.e., BF = 2(logLH_dependent_ – logLH_independent_)). We evaluated the robustness of our results by preforming the analysis with several phylogenies and three different priors (see SI Correlated evolution).

### FREQUENT ORIGINS OF HYPODERMIC INSEMINATION

We conducted ASR of the inferred mating syndrome and three proxies (received sperm location, sperm bristle state, and antrum state). First, we used the binary scorings (see SI Morphology) used in the tests for correlated evolution. However, because we expected that losses/reductions of some traits would transition via an intermediate state, we also performed ASR of the inferred mating syndrome, received sperm location, and sperm bristle state scored as trinary states. We conducted ASR using stochastic character mapping (Bollback 2006) with the R package phytools (Revell 2012). We determined the appropriate transition matrix for ASR by fitting MK‐models with equal rates (ER) of state transitions, with symmetric rates (SYM), with all rates different (ARD), and with a model without the possibility of gains once the trait is lost (Dollo). For traits with trinary states, we additionally fit an ordered model, where transitions are forced through an intermediate state (ORD) and an ordered model with no gains once the trait is lost, but allowing reversions from the intermediate state (ORD‐Dollo). We conducted ASR for models with a corrected AIC weight >0.15 (Table 2) and used the Bayesian implementation of stochastic character mapping with a gamma prior throughout (α = 1, β = 1, i.e., a low rate of transitions) and reconstructed 1000 histories (10,000 iterations burn‐in followed by 10,000 iterations and retaining every 10th character history). We summarized the number of transitions as the average number of changes as well as the 95% credible interval.

**Table 2:** Ancestral state reconstructions of reproductive traits, including received sperm location, sperm bristle state, antrum state, and inferred mating syndrome. A range of MK‐models (ER: equal rate, SYM: symmetrical rate, ORD‐Dollo: ordered model without gains once the trait is in state 0, Dollo: model without gains, ORD: ordered model, ARD: all rates different) were compared based on their AIC weights. For each trait, the model with the highest AICc weight (AICcw) is shown in bold type, but we estimated the number of transitions between the states using stochastic character mapping with 1000 posterior samples for all models with an AICc weight >0.15. Given are the average number of transitions and the 2.5% and 97.5% quantiles in brackets. Results are based on the C‐IQ‐TREE phylogeny. For the quantitatively similar results with the H‐IQ‐TREE and H‐ExaBayes phylogenies, see Table S4

Reproductive trait	Model	dfs	log lik	AICc	ΔAICc	AICcw	*N*_Species_	*N*_Changes_	0 →1	1→0	1→2	2→1	0→2	2→0
Rec. sperm location 0: hypodermic only 1: hypodermic and in antrum 2: in antrum only	ER	1	–43.3	88.6	10.1	0.004								
	SYM	3	–40.9	88.1	9.6	0.005								
	**ORD‐Dollo**	**3**	**–36.1**	**78.5**	**0**	**0.579**	**102**	**21.2 (16, 32)**	**–**	**8.0 (7, 13)**	**4.0 (0, 11)**	**9.3 (7, 13)**	**–**	**–**
	Dollo	4	–36.1	80.6	2.2	0.196	102	10.8 (9, 17)	–	0.8 (0, 4)	0.9 (0, 4)	2.6 (2, 5)	–	6.5 (4, 8)
	ORD	4	–36.1	80.6	2.2	0.196	102	23.4 (16, 38)	0.7 (0, 4)	8.4 (6, 13)	4.8 (0, 15)	9.6 (7, 15)	–	–
	ARD	6	–36.1	85.1	6.6	0.021								
Rec. sperm location 0: hypodermic 1: in antrum only	ER	1	–36.8	75.6	3.1	0.135								
	**Dollo**	**1**	**–35.2**	**72.5**	**0**	**0.638**	**102**	**9.3 (9, 11)**	**–**	**9.3 (9, 11)**				
	ARD	2	–35.2	74.5	2.1	0.227	102	9.9 (9, 14)	0.9 (0, 4)	9.0 (8, 12)				
Sperm bristle state 0: absent 1: reduced 2: present	ER	1	–82.9	167.8	27.2	0								
	SYM	3	–81.7	169.5	28.9	0								
	**ORD‐Dollo**	**3**	**–67.2**	**140.6**	**0**	**0.578**	**131**	**36.3 (29, 48)**	**–**	**12.2 (11, 17)**	**6.7 (1, 16)**	**17.5 (14, 23)**	**–**	**–**
	Dollo	4	–67.2	142.7	2.1	0.2	131	29.3 (22, 41)	–	7.1 (3, 13)	5.3 (1, 13)	12.4 (8, 18)	–	4.6 (0, 8)
	ORD	4	–67.2	142.7	2.1	0.199	131	37.1 (29, 50)	0.8 (0, 5)	13.0 (10, 20)	6.0 (1, 14)	17.3 (14, 22)	–	–
	ARD	6	–67.2	147.1	6.5	0.023								
Sperm bristle state 0: absent or reduced 1: present	ER	1	–57.6	117.3	5.7	0.038								
	**Dollo**	**1**	**–54.8**	**111.6**	**0**	**0.635**	**131**	**18.8 (18, 22)**	**–**	**18.8 (18, 22)**				
	ARD	2	–54.4	112.9	1.3	0.327	131	20.5 (17, 29)	2.7 (0, 9)	17.8 (15, 23)				
Antrum state 0: simple 1: thickened	ER	1	–48.1	98.2	3.8	0.086								
	**Dollo**	**1**	**–46.2**	**94.4**	**0**	**0.577**	**127**	**14.7 (14, 17)**	**–**	**14.7 (14, 17)**				
	ARD	2	–45.7	95.5	1.1	0.337	127	15.2 (13, 21)	2.1 (0, 6)	13.1 (11, 16)				
Inf. mating syndrome 0: hypodermic 1: intermediate 2: reciprocal	ER	1	–59.3	120.6	16.2	0								
	SYM	3	–54.4	114.9	10.4	0.003								
	**ORD‐Dollo**	**3**	**–49.1**	**104.5**	**0**	**0.578**	**119**	**34.1 (27, 47)**	**–**	**13.7 (12, 18)**	**7.1 (2, 16)**	**13.2 (10, 19)**	**–**	**–**
	Dollo	4	–49.1	106.6	2.1	0.199	119	16.6 (14, 25)	–	0.7 (0, 5)	1.1 (0, 6)	2.8 (2, 6)	–	11.9 (9, 15)
	ORD	4	–49.1	106.6	2.1	0.198	119	35.4 (27, 50)	1.0 (0, 5)	14.3 (11, 20)	7.2 (2, 16)	12.9 (9, 18)	–	–
	ARD	6	–49.1	111	6.5	0.022								
Inf. mating syndrome 0: hypodermic and intermediate 1: reciprocal	ER	1	–49.4	100.9	14.675	0.001								
	**Dollo**	**1**	**–42.1**	**86.26**	**0**	**0.997**	**119**	**14.7 (14, 17)**	**–**	**14.7 (14, 17)**				
	ARD	2	–47.3	98.68	12.421	0.002								

### CONVERGENCE IN MORPHOSPACE

We conducted a multivariate analysis to investigate whether the convergent evolution of HI is associated with changes in a variety of reproductive traits. Specifically, we summarized data on stylet, sperm, and antrum morphology (including both quantitative and categorical data) and body size using principal component analysis (15 traits, Fig. 4A; see also Fig. 1B–D). Because regular principal component analysis assumes independence of observations, an assumption violated by the phylogenetic relationships of species (Revell 2009), we calculated phylogenetically corrected principal components (pPCAs), using the phyPCA function in phytools with the lambda model. Because we combined data with different scales, we used the correlation matrix for all calculations. When discussing loadings of principal components, we apply an aggressive threshold of ±0.5, because although this results in erosion of power, it keeps false‐positive rate within expectations (Peres‐Neto et al. 2003).

### HYPODERMIC INSEMINATION AND SPERM LENGTH

To test the influence of HI on sperm length, we performed phylogenetically corrected ordinary least squared regression (PGLS) with the *gls* function in the R package *nlme* (version 3.1). We used *gls* because it allowed us to simultaneously incorporate phylogenetic signal in the residuals and account for variation in the number of measured specimens by using the sample size of the response as weights. We used log_10_‐transformed sperm length as the response variable and fit a PGLS regression for each of the binary traits included in the test of correlated evolution because they all are strong indicators of HI. Moreover, we also fit a model with the inferred mating syndrome as the predictor but coded it as binary (hypodermic and reciprocal), including the intermediate syndrome with the hypodermic syndrome. Because sperm length could be correlated with body size, we also repeated these analyses while including body size as a covariate (although note that body size did not differ between the syndromes; data not shown). We determined the best‐fitting evolutionary model for the covariance in the residuals by comparing corrected AIC of PGLS fitted with Brownian motion, lambda, or Ornstein‐Uhlenbeck models. We assessed if the assumptions of the PGLS were met by checking the distributions of the phylogeny‐corrected residuals for normality and profiled the likelihood of the parameter of the correlation structure (i.e., lambda or alpha). Because *R*‐squared values are problematic for PGLS models (Ives 2019), we evaluated model fits by calculating *R*_pred_ using the R package rr2 (Ives and Li 2018). Specifically, we compared an intercept‐only model (*Y* ∼ 1 + ε) to the full model including the predictors and the phylogeny (*Y* ∼ *X_i_* + *X_j_* + ε | Ψ) and give this value in the figures. We additionally compared the full model to a phylogeny‐only model (*Y* ∼ 1 + ε | Ψ) to determine the effect of the data after controlling for the phylogeny and give this value in the tables of the Supporting Information.

### MALE‐FEMALE COEVOLUTION

To investigate coevolution between male and female genital traits, we independently summarized five male (stylet length, stylet curviness, width of the proximal opening, width of the distal opening, and distal asymmetry, Fig. 1B) and four female traits (antrum thickness, presence and thickness of an anterior cellular valve, antrum chamber complexity, and number of genital openings; Fig. 1C) using pPCA. We then fit a PGLS regression between the first principal component of the antrum traits (antrum PC1) and the first principal component of the stylet traits (stylet PC1). And we evaluated if the lower variability of genital traits in hypodermic species could influence the result by fitting a second model that excluded hypodermic species.

## Results

### CORRELATED EVOLUTION

**Figure 3:**
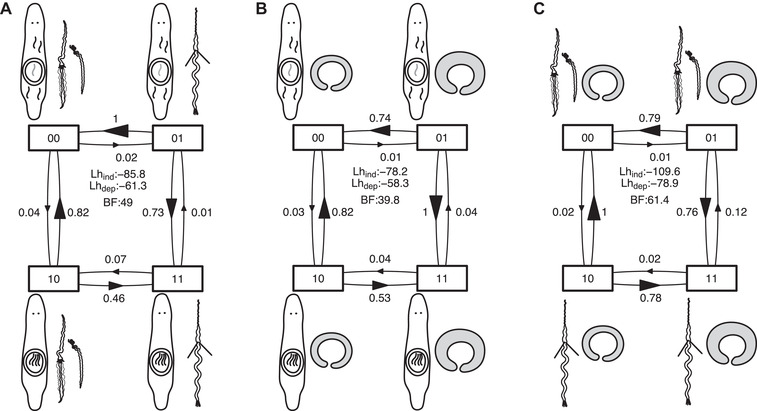
Results of correlated evolution analysis between (A) received sperm location and sperm bristle state, (B) received sperm location and antrum state, and (C) sperm bristle state and antrum state. Shown is the transition matrix for the dependent model from BayesTraits analysis, which was always preferred over the independent model. Transition rates are scaled so that the largest is unity (and arrow sizes are proportional). Also given are the likelihoods of the independent (Lh_ind_) and dependent (Lh_dep_) models, and the resulting Bayes factors (BF). An exponential prior and the C‐IQ‐TREE phylogeny was used for the results shown here. See SI Correlated evolution for runs with other priors (uniform and reversible‐jump hyperprior) and other phylogenies (H‐IQ‐TREE and H‐ExaBayes), which show qualitatively similar results.

We found strong support for correlated evolution of received sperm location with both sperm bristle state and antrum state (Fig. 3A + B). This supports previous findings that HI is associated with changes in sperm design and antrum simplification (Schärer et al. 2011). Therefore, when observations of received sperm are missing, both sperm bristle state and antrum state are likely good proxies for the mating syndrome. We expand on the earlier analyses by also providing evidence for the correlated evolution between the sperm bristle state and antrum state (Fig. 3C), which was previously implied but not formally tested (Schärer et al. 2011). Across the board, we find substantially stronger support for correlated evolution than in Schärer et al. (2011), with Bayes factors that are nearly sevenfold larger, reflecting the larger sample sizes and the larger number of transitions. Moreover, these analyses were robust with respect to the phylogeny and the priors used (see SI Correlated evolution).

### FREQUENT ORIGINS OF HYPODERMIC INSEMINATION

All ASRs indicated frequent origins of HI (Table 2 and Fig. S1). In all analyses with trinary states, an ordered transition model without gains once traits have been lost (ORD‐Dollo) was preferred, and in all analyses with binary states, a model without gains (Dollo) was preferred. However, other models, including some permitting gains, also received at least some support (Table 2). ASR of trinary states inferred frequent transitions to the intermediate state, which were driven by the ordered model's requirements to transition through it. These transitions were often placed along internal branches of the phylogeny, primarily within the clade containing *M. finnlandense*, which contains several species with reduced or absent states and, nested within them, two species with present states (*M*. sp. 12 and *M*. sp. 44, with received sperm in the antrum, long bristles, and assigned to the reciprocal mating syndrome; Fig. S1A, C, F). To represent this diversity, Figure S2 combines our Figure 2 with drawings of stylet and sperm morphology available from Brand et al. (2022).

We estimated a lower bound for the number of transitions by requiring an origin of the derived state to be separated by other such origins via nodes with a >95% posterior probability of having the ancestral state. Applying this rule to traits scored as binary, we find nine transitions to hypodermic received sperm, 18 losses/reductions of sperm bristles, 14 simplifications of the antrum, and 14 transitions to the hypodermic or intermediate mating syndrome (see red stars and numbers in Fig. S1). Moreover, these lower bound estimates were slightly lower for trinary states. Finally, we found qualitatively similar results on the other two phylogenies included, albeit, because they contain fewer species, showing fewer transitions (Table S4).

### CONVERGENCE IN MORPHOSPACE

**Figure 4:**
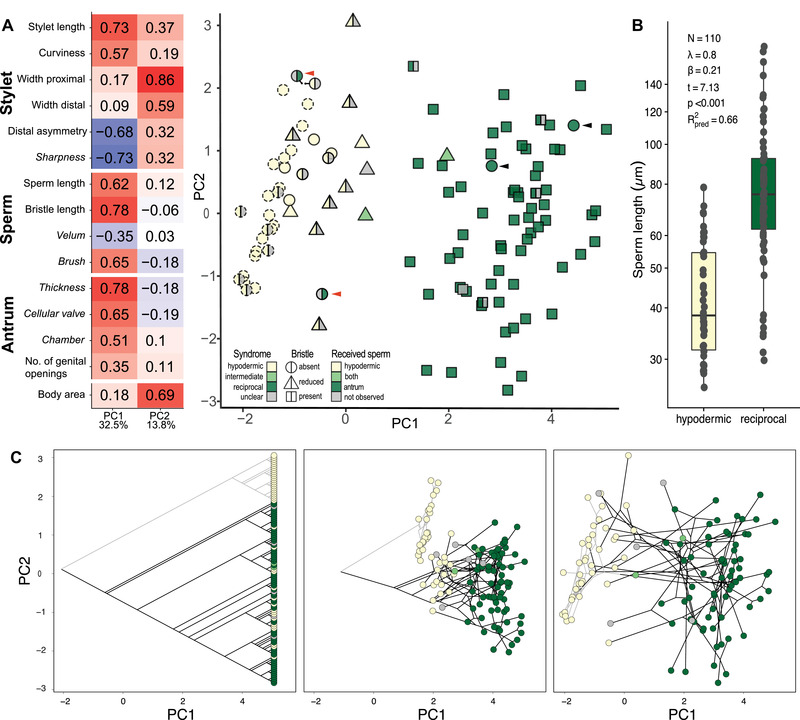
Results of a phylogenetically corrected principal component (pPCA) analysis of the measured quantitative (regular) and categorical (italics) reproductive traits (A, C) and PGLS regression of sperm length dependent on the inferred mating syndrome (B). (A) Left: Loadings of PC1 and PC2, with the percentage of variance explained at the bottom. Right: Two‐dimensional morphospace defined by PC1 and PC2. As indicated by the legend, the shape represents the sperm bristle state, whereas the colors represent the inferred mating syndrome (left side) and the received sperm location (right side). All species from the hypodermic clade are shown with stippled outlines. Red arrowheads indicate two species (*Macrostomum* sp. 51 and *M*. sp. 89) that cluster closely with species assigned to the hypodermic mating syndrome, but in which we observed received sperm in the antrum. Black arrowheads indicate two species (*M*. sp. 68 and *M*. sp. 82) assigned to the reciprocal syndrome, which have no discernible sperm bristles (see also Fig. 5, *N* = 113). (B) Sperm length of species dependent on the inferred mating syndrome. Values are slightly jittered in the *x* direction, and the *y*‐axis is on a log‐scale. Within the panel, the main results of PGLS analysis are given, with the slope being significant at p < 0.001. Results shown here are based on C‐IQ‐TREE, whereas detailed results including analyses with other phylogenies (H‐IQ‐TREE and H‐ExaBayes) are given in Table S6A. (C) The phylogenetic relationships of all species included in the pPCA analysis is represented in the left panel, and the right panel illustrates how species assigned to the hypodermic mating syndrome cluster in morphospace (as also seen in A). Edges of the hypodermic clade are printed in gray to aid in visualization. The central panel shows an intermediate state in a phytools (Revell 2012) phylomorphospace animation converting the left to the right panels (see animation in Fig. S3).

The phylogenetically corrected principal component analysis (pPCA) of all reproductive traits showed that these convergent transitions to HI coincided with changes in a larger set of reproductive traits (see also Fig. 1B–D and SI Morphology). The first two principal components, PC1 and PC2, captured nearly half of the variation in the reproductive traits (Fig. 4A), followed by additional principal components with relatively small contributions (Table S5). Specifically, PC1 captured a change in stylet phenotype, with larger values indicating species with longer, more curved stylets that are distally more symmetric and less sharp (Fig. 4A). Larger values of PC1 also indicated both longer sperm and bristles, and an increased probability for the sperm to carry a brush. Finally, high values of PC1 indicated a thickened antrum with a more pronounced cellular valve, and a more complex internal structure. In comparison, PC2 had a less clear interpretation, with high values indicating larger species with larger proximal and distal stylet openings.

**Figure 5:**
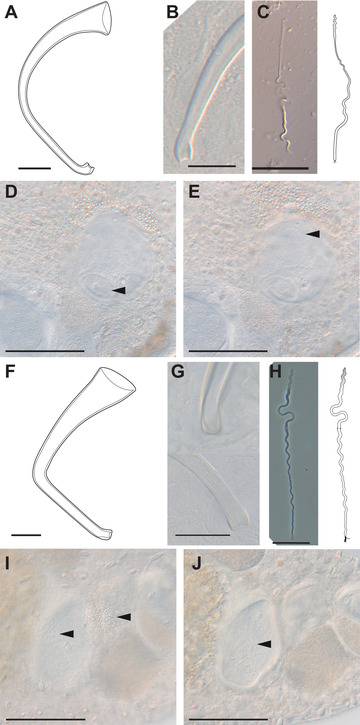
Details on the reproductive morphology of *Macrostomum* sp. 68 and *M. *sp. 82. (A‐E) *M*. sp. 68 (A) Stylet drawing showing the blunt distal thickenings; (B) distal stylet tip in a smash preparation (specimen ID MTP LS 2611). (C) Sperm image (MTP LS 2686) and drawing showing what seems to be a long feeler, but no apparent sperm bristles. (D‐E) Details of the antrum (MTP LS 2562) indicating the muscular connection between the female genital opening and the antrum (arrowhead in D) and the anterior second chamber containing at least one received sperm (arrowhead in E). (F‐J) *Macrostomum *sp. 82. (F) Drawing of the stylet showing the slight blunt distal thickenings. (G) Distal stylet tip in situ (top, MTP LS 2845) and in a smash preparation (bottom, MTP LS 2846). (H) Sperm image (MTP LS 2877) and drawing indicating the modified anterior part of the sperm (shaded gray) and a less dense area approximately one‐third along the sperm, which could be a vestigial bristle anchor location (arrowhead). (I‐J) Details of the antrum (MTP LS 2848) indicating the anterior genital opening, the bursa pore (I, left arrowhead) next to the posterior genital opening and the gonopore (I, right arrowhead), both connecting into a large chamber containing many received sperm (J, arrowhead). Scale bars represent 100 μm in the antrum images and 20 μm otherwise.

Species in the hypodermic clade (stippled outlines) had similar values in PC1 and mainly differed in PC2 (Fig. 4A). Interestingly, species from the reciprocal clade (solid outlines) that we had assigned to the hypodermic mating syndrome (left yellow) grouped closely with the species in the hypodermic clade, indicating striking convergence in morphospace concerning stylet, sperm, and antrum morphology (see Fig. 4C and animation in Fig. S3). PC1 further separated species based on the received sperm location, with hypodermic received sperm (right yellow) only found in species with low PC1, indicating that low PC1 captures a morphology necessary for HI. Almost all species with reduced (triangles) or absent (circles) sperm bristles grouped closely together in PC1, with the notable exception of *M*. sp. 68 and *M*. sp. 82 (black arrowheads), which cluster together with other species that we assigned to the reciprocal mating syndrome. We observed sperm in the antrum of both species (i.e., in two of seven specimens in *M*. sp. 68 and 16 of 21 specimens in *M*. sp. 82) and the antrum is similar in both, with a long muscular duct that performs a 90° turn toward the anterior before it enters a strongly muscular second chamber. Moreover, both species have a similar L‐shaped stylet with a blunt tip, which makes it unlikely that they mate through HI (see Fig. 5 and *Discussion*).

### HYPODERMIC INSEMINATION AND SPERM LENGTH

In addition to the changes in sperm design mentioned above, we found that HI was associated with shorter sperm. In all PGLS analyses, the states indicating the hypodermic mating syndrome were associated with shorter sperm, with the largest effect for the antrum state, followed by the inferred mating syndrome (Figs. 4B and S4; Table S6A). This is reasonable, because the bristle type falsely classified *M*. sp. 68 and *M*. sp. 82 as hypodermically mating (see *Discussion* and Fig. 5), whereas the received sperm location and inferred mating syndrome analyses had lower samples sizes. The predictive value of these PGLS models was generally high, indicating that a large proportion of the variation in sperm length is explained by the phylogeny and these indicators of the mating syndrome (Table S6A). Moreover, we also recovered similar results when we included body size as a covariate (Table S6B), which was also found to be a (marginally) positive predictor of sperm length in both the antrum state and inferred mating syndrome analyses. But including body size did not substantially increase the predictive value of these PGLS models (Table S6). Note that despite the strong association with the inferred mating syndrome, there is considerable overlap in sperm length between the species exhibiting the different states, with some species with the reciprocal mating syndrome having short sperm (Fig. 4B; Table S3) and an overall 6.7‐fold variation in sperm length across all species (with means ranging from 25.6 to 173.1 μm).

### MALE‐FEMALE COEVOLUTION

**Figure 6:**
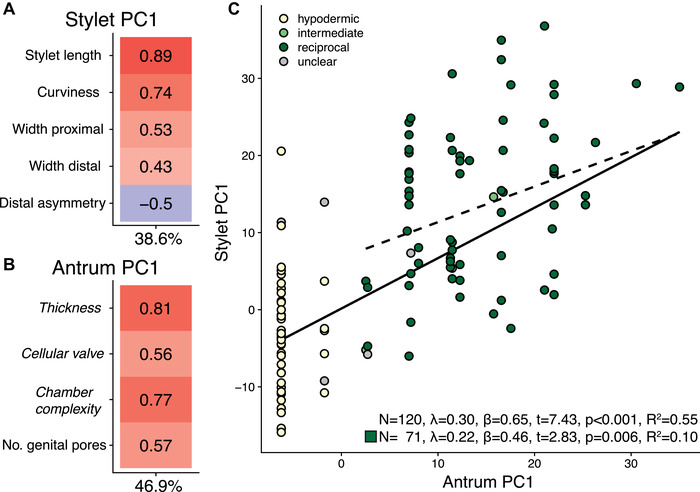
Phylogenetically corrected principal component analyses (pPCA) of stylet and antrum traits, and evidence for male‐female coevolution. (A and B) Loadings of Stylet PC1 and Antrum PC1, with the percentage of variance explained at the bottom, for the stylet traits (A) and antrum (B) traits, respectively (categorical reproductive traits are in italics). (C) Results from PGLS regression of Stylet PC1 on Antrum PC1 from (A and B). Regression was performed across all species (solid line, upper statistics, *N* = 120) and restricted to species of the reciprocal mating syndrome (dashed line, lower statistics, *N* = 71). Dot color indicates the inferred mating syndrome that the species are assigned to: hypodermic (yellow), intermediate (light green), reciprocal (green), and unclear (gray). Results based on C‐IQ‐TREE phylogeny; detailed results including analysis with other phylogenies (H‐IQ‐TREE and H‐ExaBayes) are in Table S7.

In the pPCA analysis of stylet traits, stylet PC1 was positively loaded with stylet length and the width of the proximal opening, and it was negatively loaded with distal asymmetry (Fig. 6A; Table S7). Therefore, high values of stylet PC1 represent a more elongate stylet with a wider proximal opening and less sharp distal opening. In the pPCA analysis of antrum traits, antrum PC1 was positively loaded with all variables (Fig. 6B), meaning that large values represent more complex female genitalia. A PGLS regression of stylet PC1 on antrum PC1 across all species revealed a significant positive relationship (Fig. 6C). This relationship closely matches the loadings on PC1 in the pPCA analysis of all reproductive traits (Fig. 4A) and could be driven by the simple antra in hypodermically mating species. However, the analysis including only species assigned to the reciprocal mating syndrome confirmed the positive relationship between stylet PC1 and antrum PC1 (Fig. 6C).

## Discussion

Across the genus *Macrostomum*, HI has evolved independently at least nine times as assessed by the location of received sperm, and at least 14 times based on the more inclusive inferred mating syndrome. According to Lange et al. (2013), 12 and 11 origins of traumatic insemination have been found in gonochorists and hermaphrodites, respectively (including the two cases previously documented in *Macrostomum*). This means that, based on an investigation of a single free‐living flatworm genus, we here approximately double the number of documented origins of HI among hermaphrodites. Because free‐living flatworm diversity remains notoriously understudied (Leasi et al. 2018), and because the majority of the included *Macrostomum* species are likely undescribed (Brand et al. 2022), it seems probable that we have not even documented all convergent origins of HI within *Macrostomum*. Moreover, three additional origins of traumatic insemination occur in the genus’ parent group (Macrostomorpha; Janssen et al. 2015), suggesting that traumatic insemination may evolve frequently there and potentially also in other groups of flatworms.

Interestingly, we find no clear evidence for reversals back to reciprocal mating once HI has arisen, because the Dollo models were preferred in all our ASRs (although alternative models sometimes also received some support; Table 2). Reciprocal copulation is the ancestral state of the reciprocal clade, but the state of the most recent common ancestor of the genus is less certain (Fig. S1), allowing for either a gain or a loss. Similarly, the clade containing *M. finnlandense* could contain either two independent losses or a single loss with a gain in *M*. sp. 12 and *M*. sp. 44 (Fig. S1). We favor the former hypothesis because shifts to HI might be predominantly unidirectional. Specifically, once copulation is lost, a reversal would presumably require both mating partners to again coordinate reciprocal mating behavior. Additionally, the antrum simplification that generally results from the evolution of HI could further hinder reversals, because copulating species have traits that presumably reduce the risk of injury (e.g., thickened antrum epithelia and stylets with blunt distal thickenings). In the absence of such traits, occasional reciprocal copulations could result in high fitness costs for both partners. In contrast, HI is presumably often unilateral, thus not requiring both partners to cooperate.

Our detailed observations of received sperm in both the antrum and embedded inside the recipient's tissues led us to categorize two species, *M*. sp. 3 and *M*. sp. 101, as intermediate between the mating syndromes (light green triangles in Fig. 4). These observations suggest that evolutionary transitions to HI occur through initial traumatic injection of sperm during canonical reciprocal copulation, possibly due to coincidental hypodermic sperm transfer during copulatory wounding (for a more detailed discussion, including drawings and images of where we observed sperm in these two species, see SI Pathways to HI). Once HI has evolved, recipients in some organisms evolve secondary female genitalia to avoid costs of wounding and regain control over the received ejaculate (Lange et al. 2013). Why this has not occurred in *Macrostomum* is unclear (see also below), but it might imply that costs of HI are generally low (possibly due to the striking regeneration ability of these flatworms; Egger et al. 2006) or that the location of insemination is too variable for the evolution of a localized novel organ.

An earlier study classified a needle‐like stylet and shorter, simpler sperm as adaptations for HI, and observed an associated simplification of the antrum, presumably because it is only used for egg laying in hypodermically mating species (Schärer et al. 2011). Their test of correlated evolution of discrete antrum, stylet, and sperm traits supported this hypothesis (Schärer et al. 2011), but it included only two independent origins of HI, with one containing a single species (*M. hystrix*). Although tests of correlated evolution supposedly correct for phylogenetic dependencies, it was recently pointed out that they can support the dependent model of evolution even with only a single (unreplicated) origin of the trait states in question (Maddison and FitzJohn 2015; Uyeda et al. 2018). Thus, although these previous findings supported correlated evolution, the evidence was not as decisive as these tests may have suggested. By sampling more convergent events, we here remedy this limitation, substantially raising our confidence in a causal link between sperm bristle and antrum state with HI (and this convergence has taxonomic implications, as we outline in more detail in SI Taxonomy). The increased sample size also enabled the pPCA analysis showing that species with HI indeed all have low values of PC1. Such low values correspond tightly to the mating syndromes described by Schärer et al. (2011) (note our slight adjustment of their definitions due to incomplete behavioral observations; Table 1), suggesting the underlying morphologies truly represent adaptations to HI. The striking convergent evolution thus clearly suggests that the origin of HI canalizes taxa both morphologically and behaviorally.

The morphological similarity of species with the hypodermic mating syndrome may point to a high rate of morphological evolution. It would be interesting to formally assess the rate of change in multivariate morphospace in relation to the different transitions to HI. However, our recent sampling campaign has revealed approximately 94 new species (see numbered species in Fig. 2), and, therefore, a large proportion of the diversity in this genus probably has yet to be sampled (Brand et al. 2022). Thus, many species that represent intermediate states in morphospace, and would allow more accurate estimation of evolutionary rates, might currently be missing. But note that we observe some intermediate states in the numerous species with reduced sperm bristles, as well as thickened antra in the species with the intermediate syndrome. Additionally, it is challenging to calibrate the *Macrostomum* phylogeny due to the lack of fossils in these soft‐bodied organisms, making it difficult to determine absolute rates of morphological change.

Besides HI and its associated traits, another example of convergent evolution in *Macrostomum* is the origin of a second female genital opening. The phylogeny suggests that a second opening has evolved three times (in *M. spiriger*, in *M. gieysztori* and its three close relatives, and in *M. paradoxum*; see Fig. 2). Additionally, *M*. *palum* could represent a fourth origin, but it currently lacks phylogenetic placement (for a more detailed discussion, see SI Female openings). In all species, the novel second opening is associated with a muscular bursa that could possibly allow cryptic female choice by ejecting sperm via muscular contractions. Such contraction occurs during the suck behavior in *M. hamatum*, a species with only a single opening, where sperm can be observed to be partially pushed out from the antrum even before the worm places its mouth on the female genital opening (P. Singh, pers. comm.). Given that the male genital morphologies of these species are indicative of reciprocal mating (Fig. 2), it seems unlikely that these second female genital opening are linked to mediating costs of HI, as was, for example, suggested for bed bugs (Reinhard et al. 2003).

Frequent convergent evolution of potential resistance traits, like a second female genital opening, or of alternative strategies, like HI, bolsters the interpretation that they resolve sexual conflict over mating rate, mating role, or both (Charnov 1979; Michiels 1998; Michiels and Newman 1998; Vizoso et al. 2010; Schärer et al. 2011, 2015). HI likely is an alternative strategy in an ongoing evolutionary chase between donor and recipient. This includes donor persistence traits, such as complex sperm with bristles (Schärer et al. 2011) and manipulative seminal fluids (Patlar et al. 2020; Weber et al. 2020), and recipient resistance traits, such as the suck behavior (Schärer et al. 2004) and complex female genitalia (Schärer et al. 2004; Vizoso et al. 2010), engaged in constant antagonistic coevolution (Charnov 1979; Michiels 1998; Lange et al. 2013; Reinhardt et al. 2015a). We find evidence for such male‐female genital coevolution, both across all species and within the species assigned to the reciprocal mating syndrome (Fig. 6). Our findings agree with other work on hermaphrodites (e.g., Koene and Schulenburg 2005; Beese et al. 2006; Anthes et al. 2008) and contribute to a growing body of evidence that male‐female coevolution is common in both hermaphrodites and gonochorists (Arnqvist and Rowe 2002b; Brennan et al. 2007; McPeek et al. 2009; Simmons and Fitzpatrick 2019). Genital coevolution is not only expected due to sexual conflict but also predicted in the context of sexual selection. Under the sexual selection perspective, we expect coevolution due to cryptic female choice, where the recipient will choose based on genital traits of the donor (Eberhard 1996). Donors are, therefore, selected to closely match their genital morphology to the selection criteria of the recipient. Under both views, the respective selective optima of these traits might differ between species, driving diversification and speciation (Arnqvist et al. 2000; Ritchie 2007). Our findings clearly document a dynamic evolutionary history of male‐female coevolution driving frequent innovations of sexual traits (Brand et al. 2022). Traumatic insemination allows donors to (temporarily) overcome pre‐ and postcopulatory choice and/or resistance mechanisms of the recipient, and results in striking convergence in morphospace (Fig. 4).

One striking convergent change we observe is that HI leads to a reduction in sperm size, for which we see three possible explanations. First, because HI avoids the recipient's genitalia, it probably reduces the scope for both cryptic female choice by the recipient (e.g., via the suck behavior) and sperm displacement/removal by competing donors. These postcopulatory mechanisms can introduce skews in sperm representation (Charnov 1996; Schärer 2009; van Velzen et al. 2009; Schärer and Pen 2013), which can result in lower levels of sperm competition compared to a “fair‐raffle” type sperm competition when sperm mix more freely (Parker 1982, 1993, 1998). In this case, HI could increase sperm competition and, if sperm size trades off with sperm number (Parker 1978, 1982, 1993), select for smaller sperm (Schärer and Janicke 2009; Schärer et al. 2011). Second, *Macrostomum* sperm is large compared to the size of the antrum, and therefore intimately interacts with its epithelium, often being partially embedded in the cellular valve with the feeler (Schärer et al. 2004, 2011; Ladurner et al. 2005; Vizoso et al. 2010), and sperm is also in close contact with rival sperm when recipients mate multiply (Janicke et al. 2013; Marie‐Orleach et al. 2016). Under such conditions of high sperm density, that is, when sperm displacement is likely (e.g., Miller and Pitnick 2002; Lüpold et al. 2012; Manier et al. 2013), sperm are predicted to be bigger compared to species in which the sperm storage organ is substantially larger than the sperm (Parker et al. 2010; Immler et al. 2011). Although under HI sperm still intimately interact with the parenchymal tissue of the partner, the “storage organ” could now include the whole body, reducing sperm‐sperm interaction and decreasing positive selection on sperm size. Therefore, transitions to HI could be similar to conditions with external fertilization, which strongly predict smaller sperm across all animals (Kahrl et al. 2021). Third, if small sperm can move more efficiently through the dense parenchymal tissue of the mating partner, then natural selection could favor a decrease in sperm size (Schärer et al. 2011). Little is known about sperm movement within the recipient's tissues, but it seems analogous to the undulating movement of the sperm body observed within the antrum (Willems et al. 2009). The three explanations are not mutually exclusive, and their relative importance might depend on the physiology, morphology, and ecology of each species.

Besides changes in sperm length, we confirm the finding of Schärer et al. (2011) that the evolution of HI involves the convergent reduction and loss of sperm bristles (Figs. 3 + 4), and we document hypodermic received sperm in species with reduced bristles, indicating that HI can precede the complete loss of bristles. The preference for an ordered model in the ASR even suggests that transitions via an intermediate state may be the rule. It is unclear if bristle loss is adaptive or whether it occurs due to relaxed selection and subsequent drift and/or pleiotropy, as proposed for other morphological traits (Houle et al. 2017; Jiang and Zhang 2020). Sperm bristles might result in costs for the donor, such as a reduced spermatogenesis rate or reduced sperm mobility in the partner's tissue (Schärer et al. 2011). Indeed, spermatogenesis of the complex sperm with bristles of *M. lignano* takes longer than the development of the simpler sperm in *M. pusillum* (6 vs. 4 days; Schärer et al. 2007; Giannakara et al. 2016; Giannakara and Ramm 2017). However, this could also be because *M. lignano* sperm is longer, as sperm length can be associated with a longer sperm development time (Pitnick et al. 1995; Pitnick 1996). Because several hypodermically mating species have reduced bristles, their cost in terms of movement might also be minimal, at least once they are relatively small. We also document species that very likely copulate reciprocally but do not have sperm bristles, suggesting that HI is not the only reason for bristle loss/reduction. From our observations, it appears that sperm is deposited deep inside the complex antrum of these species (Fig. 5), so that sperm bristles may no longer be necessary to resist the suck behavior (note, however, that this behavior was not seen in mating observations of *M*. sp. 82 and we currently have no mating observations of *M*. sp. 68, P. Singh, pers. comm.).

The sperm of a member of the *M. pusillum* species complex in the hypodermic clade contains electron‐dense bodies (Rohde and Faubel 1997), similar to the bristle anchor structures identified in the reciprocally mating *M. tuba* and *M. lignano* (Rohde and Watson 1991; Willems et al. 2009). If these structures are indeed remnants of sperm bristles, this would support the hypothesis (in agreement with our ASR) that bristles are symplesiomorphic in *Macrostomum*, with bristle loss as the derived condition. Moreover, sperm bristles have not been observed in three species of *Psammomacrostomum* (pers. obs.), the sister taxon of *Macrostomum* (Janssen et al. 2015), or in a presumably closely associated genus (i.e., *Dunwichia*; Faubel et al. 1994). Sperm bristles thus appear to be a novel trait that is restricted to the genus *Macrostomum*, but detailed investigations of sperm ultrastructure across the Macrostomorpha are needed to evaluate this hypothesis.

Even though sperm morphology and sperm design is exceptionally diverse across animals, little is known about the functional significance of this diversity (Birkhead et al. 2009). Because traumatic insemination originates frequently, it offers an exciting opportunity to elucidate the relative importance of natural and sexual selection for the evolution of sperm morphology (e.g., survival during sperm storage vs. rapid and efficient movement through tissue) and contribute to an integrative view of sperm ecology (Reinhardt et al. 2015b). To disentangle mechanisms shaping sperm length evolution, we should ideally investigate the sperm morphology of other groups of organisms that have evolved traumatic insemination and make use of natural variation in the location of sperm injection and sperm storage. For example, in bedbugs, the elaboration of the sperm receiving organ varies considerably from just being a slightly thickened epithelium to a complex spermalege (Hoogstraal and Usinger 1967; Siva‐Jothy 2006). If movement efficiency is a crucial constraint, we might expect a negative correlation between sperm length and tissue transit time. Also of interest are comparative investigations of sperm length in species with traumatic insemination directly into the recipient's reproductive tract, because here movement through tissue is absent and presumably other factors related to sexual selection dominate. Particularly promising in this regard would be the fly *Drosophila parabipectinata* (Kamimura 2007; but note that the presence of traumatic insemination in this clade has recently been questioned; Polak and McEvey 2021) or the spider *Harpactea sadistica* (Milan 2009).

In summary, our work clearly highlights that the genus *Macrostomum* is a promising taxon for the study of sperm form and function, combining a high morphological diversity with a large number of evolutionary origins. Additionally, these worms have desirable laboratory characteristics and a broad range of genetic tools are available (Wudarski et al. 2017, 2020; Brand et al. 2020). *Macrostomum* will also afford more in‐depth investigation of HI and shed light on this intriguing behavior's origin and function.

## Funding information

This work was supported by Swiss National Science Foundation (SNSF) research grants 31003A_162543 and 310030_184916 to LS.

## DATA ARCHIVING

The data that support the findings of this study are available in the Supporting Information of this article and openly available in Zenodo at http://doi.org/10.5281/zenodo.5702675.

## AUTHOR CONTRIBUTIONS

JNB conceptualized the idea of the study; curated the data; performed formal analysis, investigation, and visualization; wrote the original draft; and reviewed and edited the manuscript. LJH designed the methodology and performed supervision. LS conceptualized the idea of the study; performed investigation and supervision; acquired funding; administered the project; provided resources; and reviewed and edited the manuscript.

## CONFLICT OF INTEREST

The authors declare no conflict of interest.

## Supporting information

Supporting information

**Figure S1**. Ancestral state reconstructions of reproductive traits using the C‐IQ‐TREE phylogeny. The trait and type of scoring (binary/trinary) is indicated at the bottom of each panel. Stochastic character mapping is summarized with pie charts representing the proportion of stochastic maps with the respective state. Shown is the reconstruction of the best‐fitting ordered model without losses. The average number of transitions is given in Table 2, while the red stars and numbers indicate the lower‐bound number of transitions that have likely occurred (i.e. separated by nodes with >95% posterior probability of the ancestral state), while acknowledging that the ancestral state of the genus is often unclear (hence the brackets).

**Figure S2**. Enhanced version of Figure 2, additionally showing drawings of stylet and sperm morphology available from Brand et al. (2022). The ultrametric phylogeny (C‐IQ‐TREE) includes all 145 species from (Brand et al. 2022) (with 77 species depicted in Fig S2A and 68 species in Fig S2B). Branch supports are ultrafast bootstraps (top, black if 100) and approximate likelihood ratio tests (bottom, grey if 100). Species without available transcriptomes that were added based on a *28S rRNA* fragment are indicated with grey branches. Two phylogenetically well‐separated clades the “hypodermic clade” thought to exclusively mate through hypodermic insemination (HI) and the “reciprocal clade” primarily mating reciprocally can be seen in A. Columns indicate the states of five reproductive traits from light to dark (i.e. yellow, light green and dark green for trinary states; or yellow and dark green for binary states; grey indicates missing data): received sperm location (hypodermic, both, in antrum), sperm bristle state (absent, reduced, present), antrum state (simple, thickened), sharpness of stylet (sharp, neutral, blunt), inferred mating syndrome (hypodermic, intermediate, reciprocal). Stylet and sperm morphology are drawn based on our live observations, except for species with underlined names, which were redrawn based on the species description (*M. acus, M. obtusa and M. sinense* from Wang 2005; *M. heyuanense and M. bicaudatum* from Sun et al. 2015; *M. chongqingense and M. zhaoqingense* from Lin et al. 2017a; *M. shiyanense and M. lankouense* from Lin et al. 2017b; *M. shenzhenense and M. qiaochengense* from Wang et al. 2017; and *M. spiriger and M. shenda* from Xin et al. 2019). The stylet of *M. sp*. 15 is not drawn to scale, the stylets of some species are drawn at half size (stylet ½), and the stylet of M. sp. 23 is not drawn since it was incomplete. Unobserved structures are marked as no observation (no obs.).

**Figure S2**. Enhanced version of Figure 2, additionally showing drawings of stylet and sperm morphology available from Brand et al. (2022). The ultrametric phylogeny (C‐IQ‐TREE) includes all 145 species from (Brand et al. 2022) (with 77 species depicted in Fig S2A and 68 species in Fig S2B). Branch supports are ultrafast bootstraps (top, black if 100) and approximate likelihood ratio tests (bottom, grey if 100). Species without available transcriptomes that were added based on a *28S rRNA* fragment are indicated with grey branches. Two phylogenetically well‐separated clades the “hypodermic clade” thought to exclusively mate through hypodermic insemination (HI) and the “reciprocal clade” primarily mating reciprocally can be seen in A. Columns indicate the states of five reproductive traits from light to dark (i.e. yellow, light green and dark green for trinary states; or yellow and dark green for binary states; grey indicates missing data): received sperm location (hypodermic, both, in antrum), sperm bristle state (absent, reduced, present), antrum state (simple, thickened), sharpness of stylet (sharp, neutral, blunt), inferred mating syndrome (hypodermic, intermediate, reciprocal). Stylet and sperm morphology are drawn based on our live observations, except for species with underlined names, which were redrawn based on the species description (*M. acus*, *M. obtusa* and *M. sinensis* from Wang 2005; *M. heyuanensis* and *M. bicaudatum* from Sun et al. 2015; *M. chongqingensis* and *M. zhaoqingensis* from Lin et al. 2017a; *M. shiyanensis* and *M. lankouensis* from Lin et al. 2017b; *M. shenzhenensis* and *M. qiaochengensis* from Wang et al. 2017; and *M. spiriger* and *M. shenda* from Xin et al. 2019). The stylet of *M*. sp. 15 is not drawn to scale, the stylets of some species are drawn at half size (stylet ½), and the stylet of *M*. sp. 23 is not drawn since it was incomplete. Unobserved structures are marked as no observation (no obs.).

**Figure S3**. Animation of the phylomorphospace represented by PC1 and PC2 of the species in the C‐IQ‐TREE phylogeny. The animation initially shows a cladogram that then gradually transforms into the phylomorphospace, which was calculated using the phylomorphospace function in phytools (Revell 2012).

**Figure S4**. Sperm length of species dependent on (A) received sperm location, (B) sperm bristle state, and (C) antrum state. Values are slightly jittered in the x direction, and the y‐axis is on a log‐scale. Within each panel the main results of a PGLS analysis are given and in all tests the slopes were significant at p <0.001. Detailed results including analyses with different phylogenies (H‐IQ‐TREE and H‐ExaBayes) are given in Table S6A.

**Table S1**. The number of specimens analysed per *Macrostomum* species for all the included quantitative traits.

**Table S2**. Details on all specimens included in this study.

**Table S3**. Mean species values for all morphological variables.

**Table S4**. Ancestral state reconstruction using stochastic character mapping.

**Table S5**. Scores and loadings from the phylogenetically corrected principal component analysis.

**Table S6**. Results of PGLS analysis of states indicating reciprocal copulation versus hypodermic insemination on sperm length. All predictors were binary, with the reference level being the state indicating hypodermic insemination.

**Table S7**. Results from PGLS correlating the first principal components of a phylogenetically corrected principal component analysis (pPCA) analysis including five stylet traits with the first principal component of a pPCA analysis including four antrum traits. Analysis was performed across all species and restricted to the reciprocal mating syndrome. Also given are pPCA loadings and results for all three phylogenies.
