# Supplementary figures and images for: Frequent origins of traumatic insemination involve convergent shifts in sperm and genital morphology
Source: Evol Lett. 2021 Dec 30;6(1):63–82. doi: 10.1002/evl3.268 (PMC8802240; doi:10.1002/evl3.268)

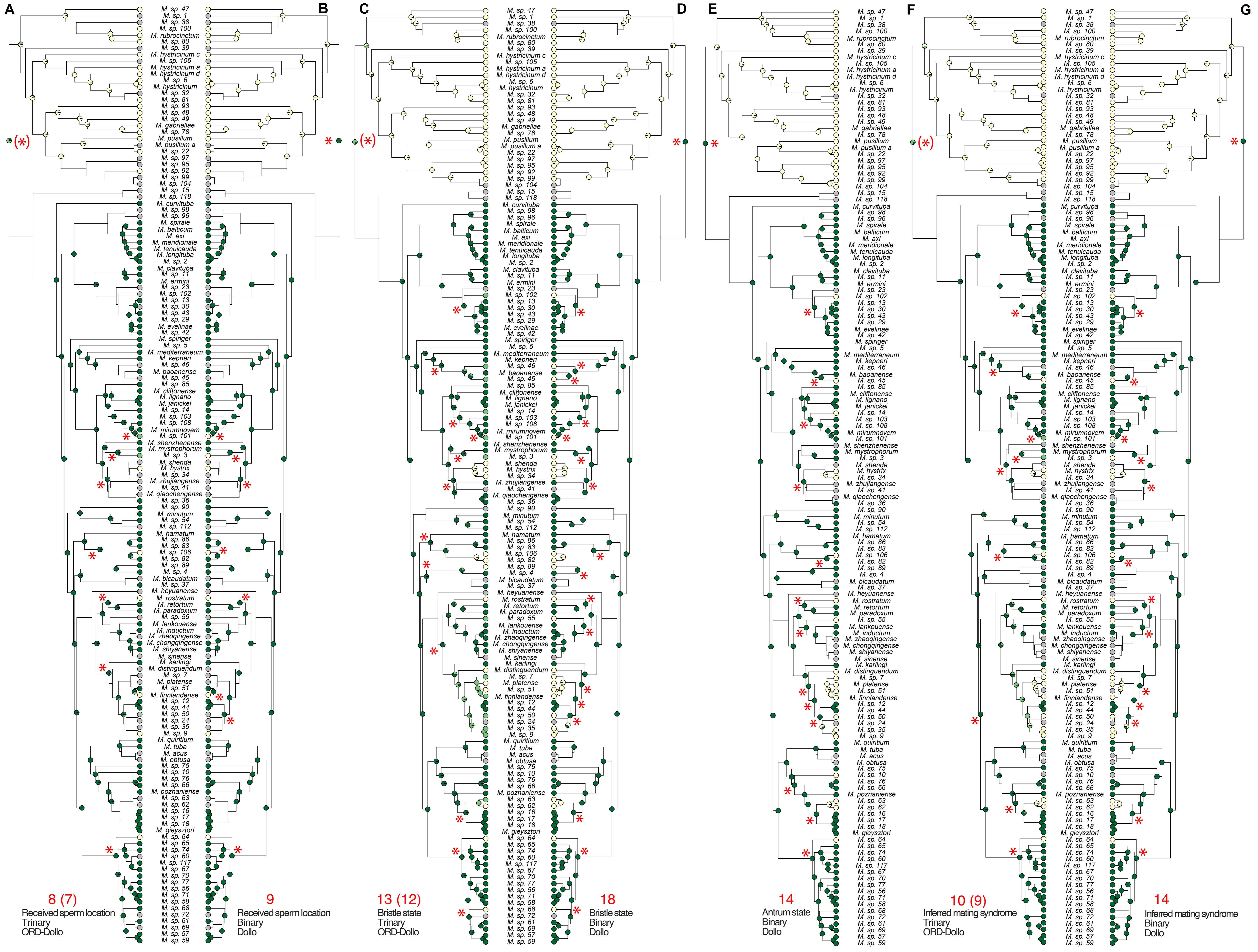

Supplement: Supplementary file 2 — Figure S1. Ancestral state reconstructions of reproductive traits using the C‐IQ‐TREE phylogeny. The trait and type of scoring (binary/trinary) is indicated at the bottom of each panel. Stochastic character mapping is summarized with pie charts representing the proportion of stochastic maps with the respective state. Shown is the reconstruction of the best‐fitting ordered model without losses. The average number of transitions is given in Table 2, while the red stars and numbers indicate the lower‐bound number of transitions that have likely occurred (i.e. separated by nodes with >95% posterior probability of the ancestral state), while acknowledging that the ancestral state of the genus is often unclear (hence the brackets). [file EVL3-6-63-s003.pdf]

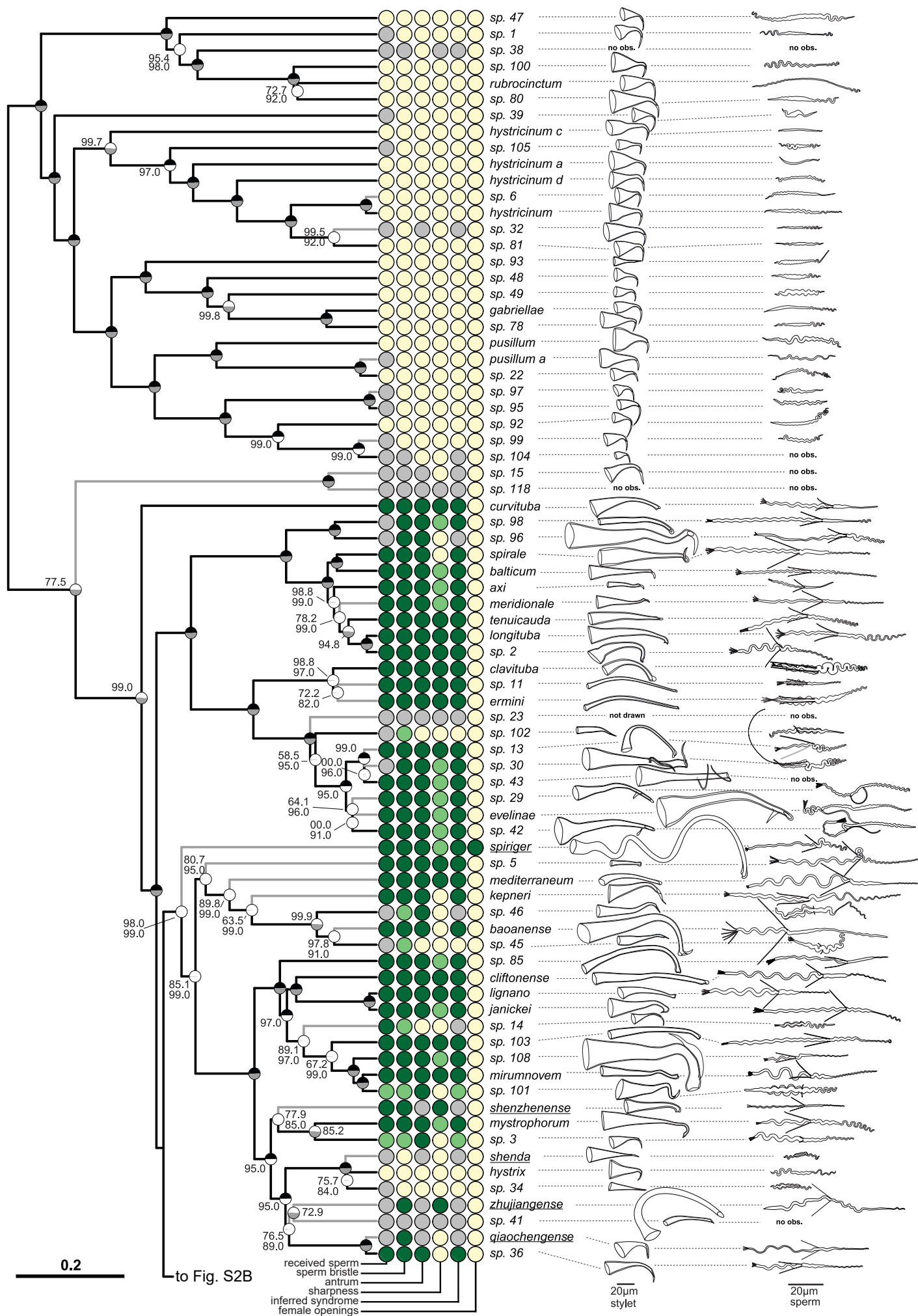

Supplement: Supplementary file 3 — Figure S2. Enhanced version of Figure 2, additionally showing drawings of stylet and sperm morphology available from Brand et al. (2022). The ultrametric phylogeny (C‐IQ‐TREE) includes all 145 species from (Brand et al. 2022) (with 77 species depicted in Fig S2A and 68 species in Fig S2B). Branch supports are ultrafast bootstraps (top, black if 100) and approximate likelihood ratio tests (bottom, grey if 100). Species without available transcriptomes that were added based on a 28S rRNA fragment are indicated with grey branches. Two phylogenetically well‐separated clades the “hypodermic clade” thought to exclusively mate through hypodermic insemination (HI) and the “reciprocal clade” primarily mating reciprocally can be seen in A. Columns indicate the states of five reproductive traits from light to dark (i.e. yellow, light green and dark green for trinary states; or yellow and dark green for binary states; grey indicates missing data): received sperm location (hypodermic, both, in antrum), sperm bristle state (absent, reduced, present), antrum state (simple, thickened), sharpness of stylet (sharp, neutral, blunt), inferred mating syndrome (hypodermic, intermediate, reciprocal). Stylet and sperm morphology are drawn based on our live observations, except for species with underlined names, which were redrawn based on the species description (M. acus, M. obtusa and M. sinense from Wang 2005; M. heyuanense and M. bicaudatum from Sun et al. 2015; M. chongqingense and M. zhaoqingense from Lin et al. 2017a; M. shiyanense and M. lankouense from Lin et al. 2017b; M. shenzhenense and M. qiaochengense from Wang et al. 2017; and M. spiriger and M. shenda from Xin et al. 2019). The stylet of M. sp. 15 is not drawn to scale, the stylets of some species are drawn at half size (stylet ½), and the stylet of M. sp. 23 is not drawn since it was incomplete. Unobserved structures are marked as no observation (no obs.). [file EVL3-6-63-s013.pdf]

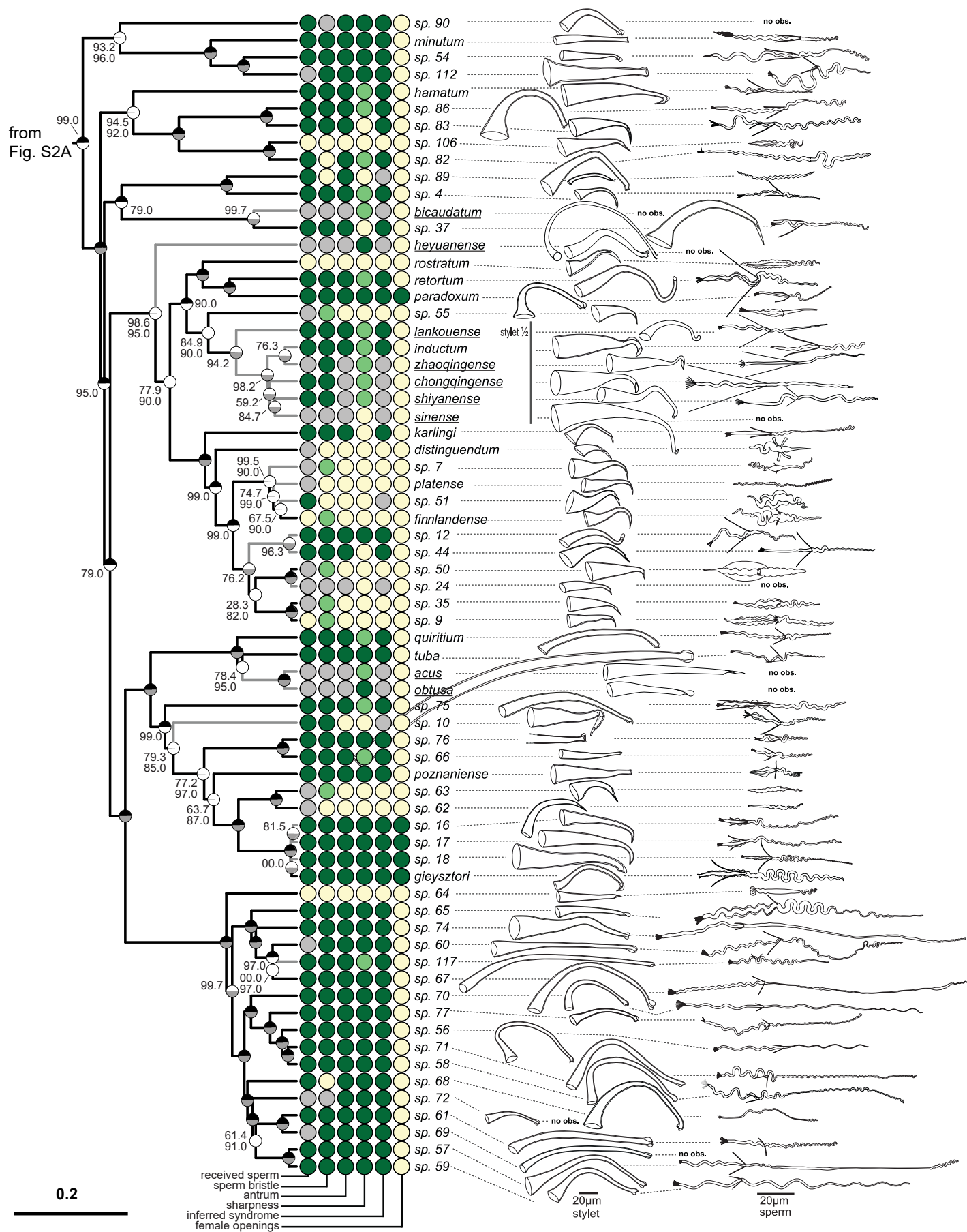

Supplement: Supplementary file 4 — Figure S2. Enhanced version of Figure 2, additionally showing drawings of stylet and sperm morphology available from Brand et al. (2022). The ultrametric phylogeny (C‐IQ‐TREE) includes all 145 species from (Brand et al. 2022) (with 77 species depicted in Fig S2A and 68 species in Fig S2B). Branch supports are ultrafast bootstraps (top, black if 100) and approximate likelihood ratio tests (bottom, grey if 100). Species without available transcriptomes that were added based on a 28S rRNA fragment are indicated with grey branches. Two phylogenetically well‐separated clades the “hypodermic clade” thought to exclusively mate through hypodermic insemination (HI) and the “reciprocal clade” primarily mating reciprocally can be seen in A. Columns indicate the states of five reproductive traits from light to dark (i.e. yellow, light green and dark green for trinary states; or yellow and dark green for binary states; grey indicates missing data): received sperm location (hypodermic, both, in antrum), sperm bristle state (absent, reduced, present), antrum state (simple, thickened), sharpness of stylet (sharp, neutral, blunt), inferred mating syndrome (hypodermic, intermediate, reciprocal). Stylet and sperm morphology are drawn based on our live observations, except for species with underlined names, which were redrawn based on the species description (M. acus, M. obtusa and M. sinensis from Wang 2005; M. heyuanensis and M. bicaudatum from Sun et al. 2015; M. chongqingensis and M. zhaoqingensis from Lin et al. 2017a; M. shiyanensis and M. lankouensis from Lin et al. 2017b; M. shenzhenensis and M. qiaochengensis from Wang et al. 2017; and M. spiriger and M. shenda from Xin et al. 2019). The stylet of M. sp. 15 is not drawn to scale, the stylets of some species are drawn at half size (stylet ½), and the stylet of M. sp. 23 is not drawn since it was incomplete. Unobserved structures are marked as no observation (no obs.). [file EVL3-6-63-s007.pdf]

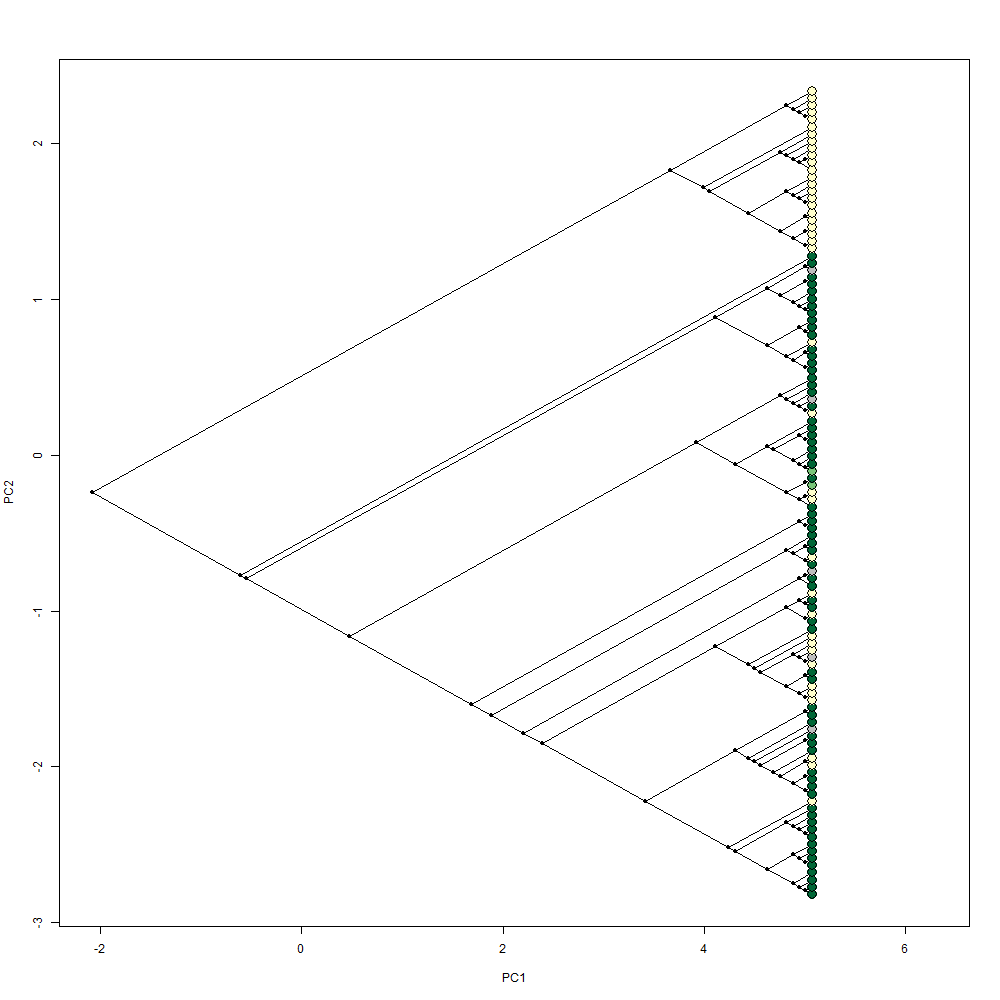

Supplement: Supplementary file 5 — Figure S3. Animation of the phylomorphospace represented by PC1 and PC2 of the species in the C‐IQ‐TREE phylogeny. The animation initially shows a cladogram that then gradually transforms into the phylomorphospace, which was calculated using the phylomorphospace function in phytools (Revell 2012). [file EVL3-6-63-s008.gif]

**A**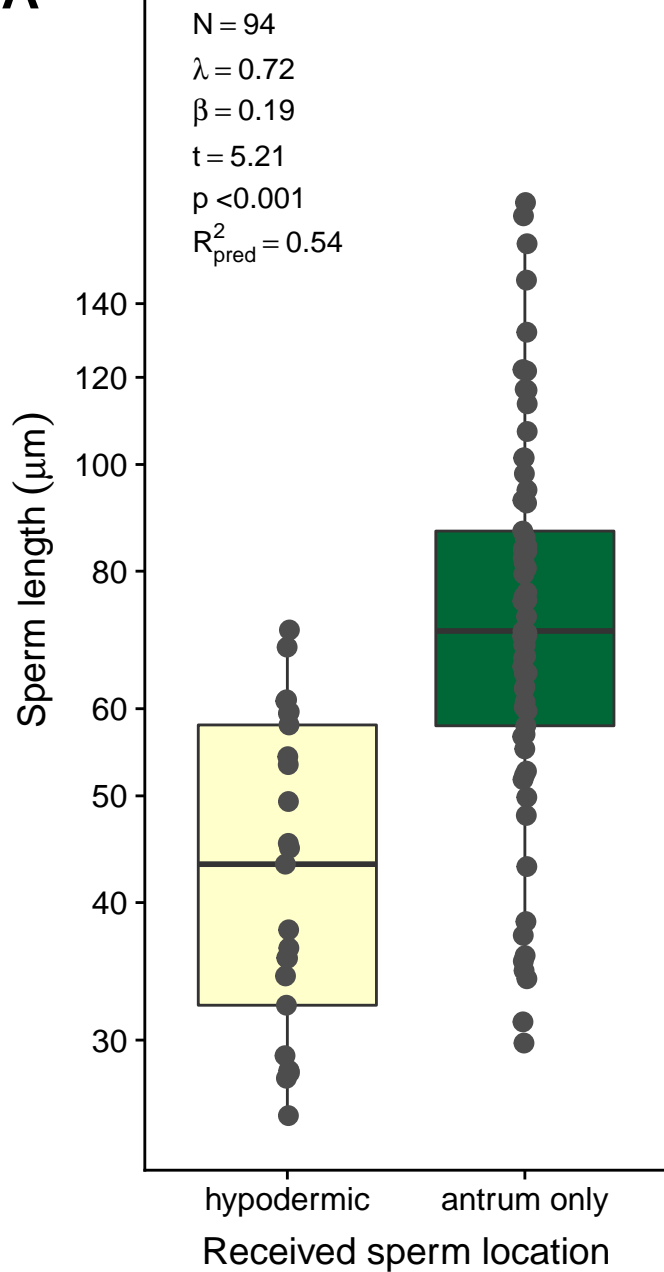**B**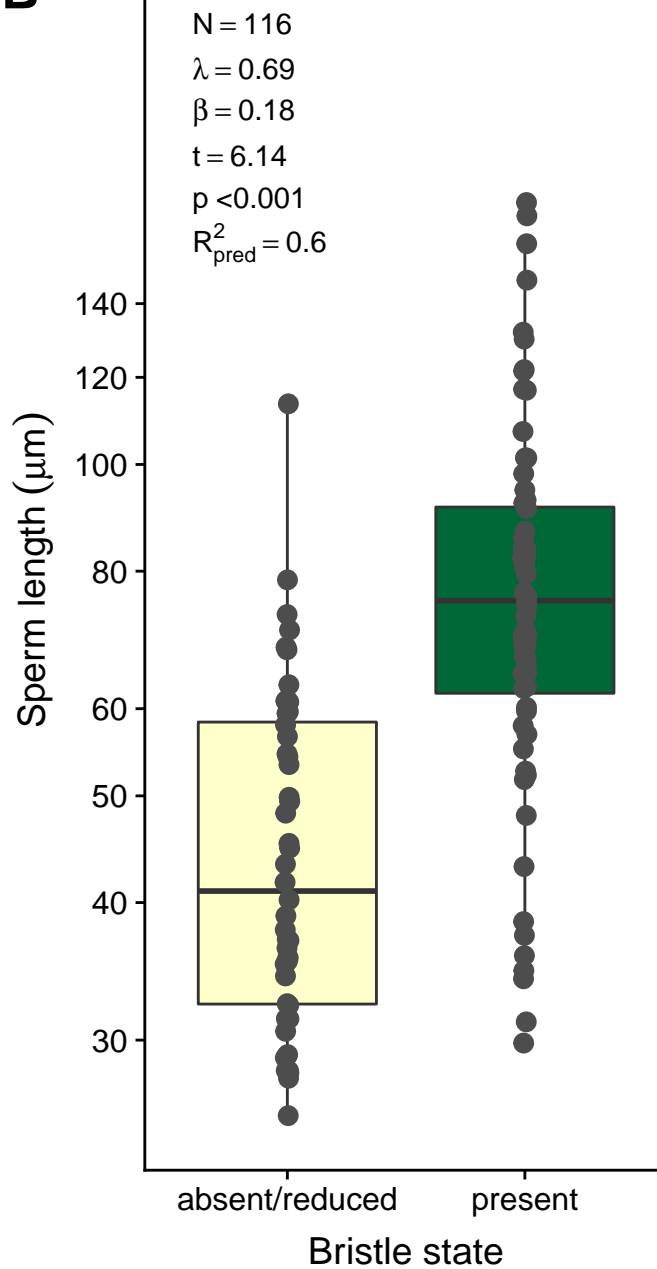**C**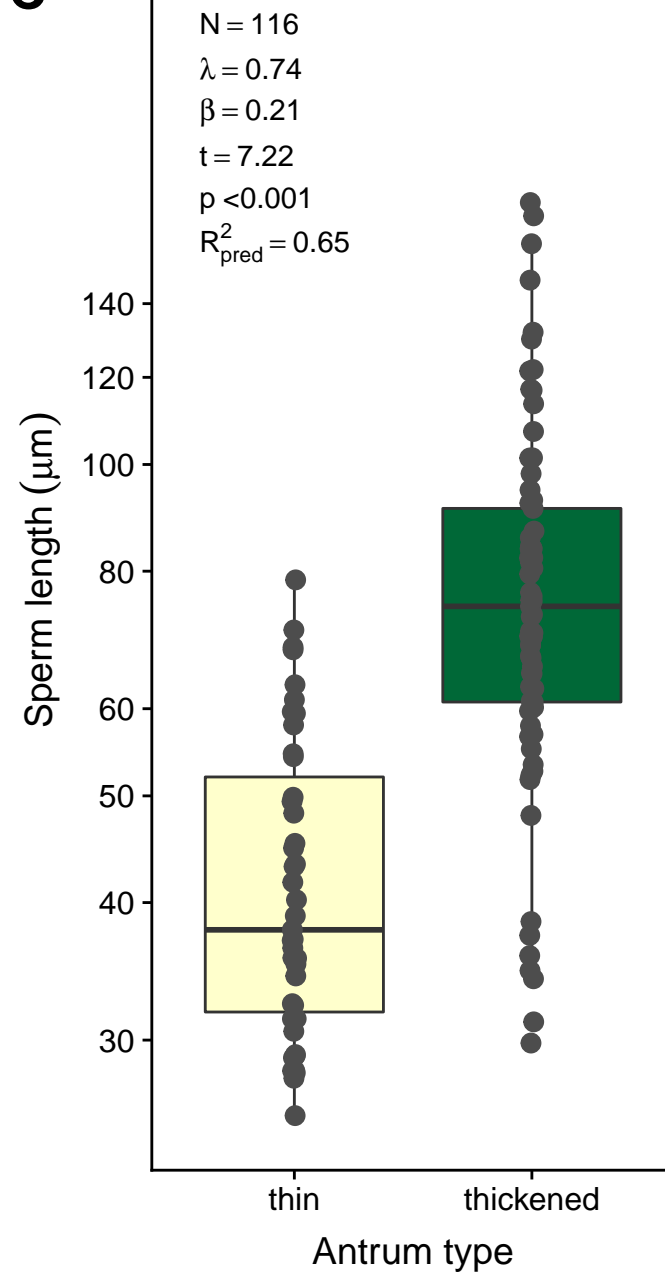

Supplement: Supplementary file 6 — Figure S4. Sperm length of species dependent on (A) received sperm location, (B) sperm bristle state, and (C) antrum state. Values are slightly jittered in the x direction, and the y‐axis is on a log‐scale. Within each panel the main results of a PGLS analysis are given and in all tests the slopes were significant at p <0.001. Detailed results including analyses with different phylogenies (H‐IQ‐TREE and H‐ExaBayes) are given in Table S6A. [file EVL3-6-63-s005.pdf]
